# The effects of listening on speaker and listener while talking about character strengths: an open science school-wide collaboration

**DOI:** 10.1098/rsos.221342

**Published:** 2024-12-18

**Authors:** Tia Moin, Netta Weinstein, Guy Itzchakov, Amanda Branson, Beth Law, Lydia Yee, Emma Pape, Rebecca Y. M. Cheung, Anthony Haffey, Bhismadev Chakrabarti, Philip Beaman

**Affiliations:** ^1^ University of Reading, Reading, UK; ^2^ University of Haifa, Haifa, Israel

**Keywords:** listening, authenticity, positivity-resonance, anxiety, strengths

## Abstract

Listening is understood to be a foundational element in practices that rely on effective conversations, but there is a gap in our understanding of what the effects of high-quality listening are on both the speaker and listener. This registered report addressed this gap by training one group of participants to listen well as speakers discuss their character strengths, allowing us to isolate the role relational listening plays in strengths-based conversations. Participants were paired and randomly assigned to a high-quality listening (experimental) or moderate-quality listening (comparison) condition manipulated through a validated video-based training. High-quality listening predicted a more constructive relational experience; specifically, positivity resonance. Intrapersonal experiences (perceived authenticity and state anxiety) were not affected. Those who engaged in high-quality listening expressed a behavioural intention to continue listening, but condition did not predict a behavioural intention for speakers to continue applying character strengths. This is the first evidence of positivity resonance as a shared outcome between both a speaker and listener when the listener conveys high-quality (as opposed to ‘everyday’) listening. These early findings merit further study with stronger listening manipulations to explore the potential role of listening within interpersonal communication, and inform the applied psychological sciences (counselling, psychotherapy, coaching, organizational, education).

## 1. Introduction

A growing literature suggests that listening well can support a deep connection and sense of wellbeing within speakers as they share important thoughts, emotions and experiences with their conversation partners, though less is known about the impact on the listeners themselves [1,2]. In this pre-registered study, we explore whether the quality of listening can enhance both speakers’ and listeners’ experiences as they discuss a topic selected to stimulate personal and consequential real-life conversations: their *character strengths*. Identifying and then using one’s character strengths—understood as ‘positive traits/capacities that are personally fulfilling, do not diminish others, [and are] ubiquitous and valued across cultures’ [3, p. 2])—increases wellbeing and buffers the negative impact of stress and psychopathology [4]. ‘Strengths spotting’ [5], an active and constructive acknowledgement [6] of strengths by others, is seen as one key factor among others in strength-based interventions [7]. No research of which we are aware examined whether the benefits of these conversations are attained as a direct function of the relational climate in which they take place, in this case, relational listening.

To fill this gap, we test the notion that high-quality listeners can aid speakers as they think about and discuss their strengths and that the listeners can also benefit from such conversations. We build on a foundation of listening effects (e.g. [8–10]) and extend this work in two ways. First, though research is accumulating about the potential benefits of listening, little evidence has emerged from experimental tests of listening, and fewer studies yet combine experimental methods with naturalistic conversations between individuals. Second, more of this work is needed to understand how the speaker, and the listener, are both impacted by their conversations. The proposed project sets out to test this paradigm using a study design and data-collection approach that involves a school-wide collaboration with academic supervisors and their students, placing the theoretical contribution on equal ground with the pedagogical benefits of testing it as a multi-lab (i.e. supervisors and their students) collaboration.

### 1.1. Listening in conversations

High-quality listening matters within many of life’s relationships. It is the bedrock of constructive psychotherapy [11,12], effective coaching [13], thoughtful parenting [14,15], engaging management [16] and effective education and supervision [17,18]. Across these interpersonal contexts, listening can be understood as a complex construct comprising three dimensions that are conveyed by the listener to the speaker [2]. First, listeners convey their careful attention to what is said by using non-verbal behaviours such as maintaining appropriate eye contact [19], body posture, and facial expressions that convey openness [20], and head-nodding [21]. Second, listeners convey their comprehension of what is said, for example by using verbal behaviours that indicate to the speakers that the listeners understand them. These behaviours include asking open questions [22] and summarising the speakers’ content (i.e. paraphrasing [23]). The third component, positive intention, refers to behaviours that convey to the speakers that their listeners are caring, non-judgmental, and want to help. Positive intention can be conveyed through the tone of the listener [24], by using ‘soft’ hedging phrases such as ‘perhaps’ or ‘might’ [25], and by providing validation (e.g. ‘thank you for sharing this with me’ [26]). While people tend to understand these complexities of listening, they also perceive listening in a holistic way [27].

A common misperception is that listeners play a passive role in the conversation, and it is the speakers who shape the tone of the conversation [20,28]. On the contrary, various studies that manipulated listening quality found that listeners’ behaviour impacts speakers’ emotions, cognitions, and behaviours. For example, listening quality relates to greater speech fluency [29], better memory of the conversation [30], greater attitude clarity [31], more helping behaviours [32] and more empowerment [33] among other potential benefits (for a detailed review on the effects of listening, see [2,34].

### 1.2. Benefits of listening on the listener

Although the brunt of this work has focused on the benefits of listening for the speaker, there is reason to expect that listening affects the *listener* alongside those effects experienced by the speaker. For example, professions such as management, teaching and particularly coaching and counselling place emphasis on listening in facilitating desired practitioner outcomes [12,35–37]. While it is understood that high-quality listening plays a key role in forming a relationship between a practitioner and speaker [38], it is less well understood how the listener experiences high-quality listening and exactly how listening facilitates outcomes. For example, taking coaching and counselling where listening is foundational to practice, it has been found that the effects on the listener can be both transformational and detrimental [9,10]. For the coach practitioner, one case study's findings revealed that performing coaching improved listening ability, as well as interpersonal skills, self-regulation (calmer and more focused as a result of better listening), confidence, sense of achievement, broadened perspective and work–life balance [8]. On the contrary, another study revealed the detrimental effects on counsellors from listening to stories about trauma and explored mental and practical strategies that could mitigate these negative outcomes [10].

### 1.3. Mechanisms for listening effects on wellbeing

In recent conceptual papers, researchers posit that conversations with high-quality listening hold benefits because they promote positive interpersonal and intrapersonal experiences during the conversation itself, which can carry weight with conversation partners [24]. We review three specific benefits that will be the focus of this work below, which can help us to understand the reactions of both speakers and listeners in a high-quality listening context: authenticity, positivity resonance and anxiety.

First, building on the episodic listening theory (hereafter ELT; 2), a conceptual approach that highlights the importance of high-quality listening for promoting speakers’ authenticity, we test the extent to which feeling authenticity is critical for the positive outcomes that speakers gain from being listened to. According to ELT, listening can even catalyze shared authenticity between conversation partners—the speaker’s authenticity supports the listener in an upward cycle.

Though authenticity is believed to be an important outcome of listening in the ELT, this has not been empirically tested. Authenticity is a concept difficult to define [39], and it is unclear whether there is actually a ‘true self’ [40]. Yet the dominant definition describes that authenticity reflects a congruence between one’s internal experience, awareness of that experience, and self-expression [41]. This aligns with the concept of authenticity that is also described within self-determination theory [42], which proposes that individuals who behave according to autonomous, intrinsic motivations feel themselves to be authentic. Importantly, in previous research, such conceptions of authenticity relate to downstream wellbeing [39,43]. Indirect evidence supporting the link between listening and authenticity comes from work on autonomy need satisfaction, operationally tested in terms of feeling free to express oneself genuinely, and feeling that one can be ‘who they are’, among other, similar experiences [42]. In previous research across several conversation types, speakers who received high-quality listening have reported greater autonomy need satisfaction than speakers who experienced moderate-quality listening [44,45].

Whereas authenticity may be a critical understudied *intrapersonal* outcome of listening, positivity resonance is an *interpersonal* outcome of listening that has received little attention in this context. *Positivity resonance* refers to a momentary interpersonal connection that is evoked between individuals [46] when individuals have an interpersonal connection characterized by shared positive affect, mutual care and concern, and behavioural and biological synchrony [46,47]. As with authenticity, there has been little attention paid to the effect of listening on positivity resonance with the exception of [48]. Supporting evidence comes from work that high-quality listening increases speakers’ sense of relatedness [49], defined as a sense of closeness to others [50]. Relevant to the listener, case studies in coaching and counselling described earlier support that those who engage in high-quality listening emotionally resonate with speakers. In non-clinical engagements such as coaching, they may be more likely to co-experience positive emotions. Indeed, co-experienced emotional experiences, particularly positive ones, have been found to support the perception of high-quality relationships and are consistent with the theory of positivity resonance [46,51], lending support to the idea that high-quality listening can facilitate a co-experienced emotional state that breeds intimacy.

Though there is reason to believe relational qualities of authenticity and positivity resonance drive downstream benefits, it may instead be that listening benefits are simply due to reduced anxiety because listeners create a relaxed and non-judgemental space in which to share ideas. This idea is not new. Carl Rogers, one of the noted fathers of modern psychology, theorized that when speakers feel listened to in a non-judgmental manner they become more relaxed and free from evaluative concerns [52]. In this work, we will define such *state anxiety* as temporary distress or physiological arousal in response to the potential for undesirable consequences [53,54]. State anxiety can also arise when people perceive a discrepancy between the reactions of others and the standard people set for themselves [55]. For example, a person may want to convey an image of an intelligent person in front of another person and is worried that they will say something that might make them appear foolish. There is mounting evidence that speakers who experience high-quality listening feel less anxiety than those who experience lower listening levels [31,56,57], but this affective mechanism has never been tested alongside interpersonal or self-based approaches. Furthermore, in relation to the listener, applied literatures describe the experience of ‘critical moments’ in the coaching interaction [58] or ‘moments of meeting’ in the therapeutic relationship [59], described as shared intense emotional experiences. These shared moments can be productive if approached in a certain way or, alternatively, they can lead to anxiety and the need for strategies to resolve difficult feelings felt by the listener [10,60]. To bridge these areas, the current study is planned to directly measure and compare the proximal downstream effects of these conversation-level outcomes of listening in the context of a conversation about character strengths.

### 1.4. Listening to character strengths

There are various conceptualizations of ‘strengths’ (e.g. [5,61,62]; overview [4]), ranging from the qualities that we have become skilled and experienced in, to virtuous, positive aspects of personality. For this study, we have chosen to focus on the latter, aligning with character strengths [61]; positive attributes that can be used to engage in life and work towards desired end goals [63]. Character strengths have been extensively researched in the last decade [64] in the context of wellbeing [65,66] and as buffers for the detrimental effects of psychopathology [67] and adverse situations [68,69]. Some literature distinguishes between having an *awareness* of strengths and *using* one’s strengths [7,70]. Meta-analytic findings link identifying and then using one’s character strengths (i.e. through strengths-based interventions) to happiness, decreased depression and life satisfaction [71] and reduced stress [72,73]. However, alongside this, recent findings reveal that awareness alone (of one’s self-perceived strengths), without applying the strengths, is sufficient to generate positive outcomes including optimism, reduced stress, and less negative wellbeing in the anxiety-producing context of taking examinations [70]. Yet, it is those with higher baseline levels of self-esteem and positive affect that reported the greatest benefits after writing down their strengths, suggesting the presence of moderators to this effect [70]. Other studies have suggested that relational experiences may shape the outcomes of talking about strengths [7,74], and we aimed to isolate the relational aspects from the character strengths in our study.

Building on previous work suggesting that identifying one’s strengths relates to greater motivation and intention to leverage one’s strengths for self-improvement and goal success [75], we also sought to determine whether the positive outcomes of high-quality listening would result in a downstream benefit, that is an intention to continue what was experienced during the conversation (exploring character strengths, or continuing high quality listening). Indeed, studies on behavioural intention support that positive experiences can lead to increased satisfaction, resulting in behavioural intention to repeat the experience (e.g. [76,77]). Building on this work we believe that the intrapersonally and interpersonally rewarding climate created by high-quality listening would shape intention to continue engaging in the activity for the listener (listening) and for the speaker (applying character strengths).

## 2. Present study

### 2.1. Theoretical aims of the project

**Figure 1.**
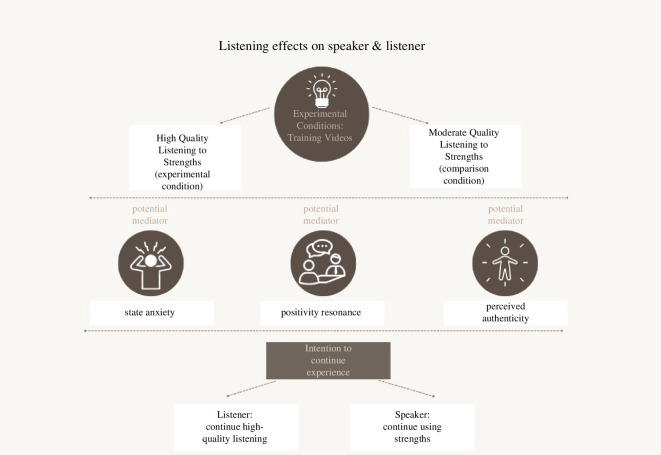
Listening effects on speaker and listener.

The project had an overarching theoretical aim: to build our understanding of the outcomes of *high-quality listening* during a positive conversation about character strengths. Further, at the time of writing, there were no studies that empirically isolate the effects of high-quality listening on the listener, and few that attempt to understand why both listeners and speakers may benefit from their conversations. This research explores the specific outcomes of listening to strengths that may apply to both speakers and listeners in a high-quality listening conversation. As an added dimension, we expected a positive benefit from the effects of high-quality listening will be a behavioural intention to continue the experience. Our hypotheses (below) relate to the proposed conceptual model depicted in figure 1.

We set out to test three directional hypotheses (*H*):

*H*_1_: Both *speakers* and *listeners* who participate in a high-quality listening conversation about character strengths would report more positive interpersonal and intrapersonal conversation experiences (positivity resonance, authenticity, lower state anxiety) than when in the control condition, where we anticipate that moderate-quality listening will take place.

*H*_2a_: *Speakers* who participate in a high-quality listening conversation about character strengths would report a greater intention to use their character strengths following the conversations.

*H*_2b_: *Listeners* who participate in a high-quality listening conversation about character strengths would report a greater intention to continue engaging in high-quality listening.

*H*_3a_: For *speakers*, the effects of condition on intention to use strengths following the conversations would be mediated by positivity resonance, authenticity and lower anxiety.

*H*_3b_: For *listeners*, the effects of condition on the intention to continue high-quality listening following the conversations would be mediated by positivity resonance, authenticity and lower state anxiety.

### 2.2. Methodological goal

Alongside the conceptual aims, we also pursued a methodological advance to build on the existing listening literature. Within the listening research, the state of the art involves staging live conversations, ideally paired with an experimental manipulation for drawing causal conclusions. This pairing is easier said than done, but two primary approaches have been attempted in past experiments. The first involves distracting listeners, for example, by placing flickering computer screens behind the speaker (e.g. [31,78]) or instructing listeners to complete a cognitive task that disrupts their ability to concentrate [29,79]. A second approach is to rely on trained confederates (e.g. [24,80]). Both approaches have their limitations. The distinction between regular and high-quality listening is important in determining the presence of psychological factors that are not detectable in low-quality or moderate listening conditions [78]. Manipulating listening by distracting listeners does not enable testing better-than-average or high-quality listening, and it might simultaneously manipulate rudeness [2]. Manipulating listening using trained confederates addresses these problems. However, such experiments are lengthy and expensive, they are not ‘natural’ conversations and are vulnerable to researcher bias. In the present study, we manipulated listening through the use of training videos (see electronic supplementary material, appendix 1), which were viewed by participants assigned to the role of listener only, paired in dyads with a speaker who watched a neutral video. This approach involves an experimental manipulation preceding conversations that unfold naturally between paired participants. It therefore benefits from relatively high internal and external validity.

### 2.3. A collaborative, pedagogical research process

The project had a tertiary aim: to undertake a collegial collaboration across an academic department that promotes new working relationships among teaching staff, educates and engages students in the value of Open Science, and leverages the benefits of cross-disciplinary collaboration in advancing research (see electronic supplementary material, appendix 2).

## 3. Method

### 3.1. Ethical approval

The research complies with the British Psychological Society ethical guidance and has been approved by the School of Psychology and Clinical Language Sciences Ethical Review Board at the University of Reading. Participants were issued with information about the study aims, procedure, commitments required of them, and data management plan upon invitation. Participants provided written consent and verbal assent prior to engagement in research activities and were reminded that they could withdraw from the study at any point. Participants were also offered an opportunity to debrief following completion. As the topic of conversation is generally positive in nature, we did not expect there to be any adverse effects from participating in the study.

### 3.2. Open research practice

Raw data, materials and time-stamped pre-registration were made available for download on the Open Science Framework following Stage One peer review but before starting data collection to comply with best practice. For study scales, and for information on the design, analysis and data incorporated recommendations made by reviewers in the first stage, please refer to the registered report on the Open Science Framework: https://osf.io/kdvx5.

## 4. Participants

### 4.1. Recruitment strategy

Participants were recruited by student researchers through snowballing procedures and by using the participant pool at the academic institution. Participants were 18 years and older, spoke English at a conversational level, and could hear speech and sound to ensure consistency in the interpretation of listening signals. Outside of this requirement, there were no other exclusion or inclusion criteria. We collected demographic data on participant ethnicity, nationality and whether participants spoke English as their home language to explore whether notions of high-quality listening differ across cultures, and whether ‘matched’ pairings differ from ‘mixed’ pairings in participant characteristics such as gender, age and ethnicity. Participants were required to have access to suitable video-conferencing facilities; where they did not have access to this, they were offered a physical space to engage in the interaction. Participants were included in a raffle to win one of three £300 prizes and undergraduate students received course credits for participating.

### 4.2. Sample sizes

Through the school collaboration, we invited all potential peer collaborators and any of their students who were interested in participating. Therefore, we could not confidently estimate our final sample size for our registered report. Furthermore, considering the novelty of the dependent variables in relation to listening effects, we recognized we could not rely on existing benchmarks for *a priori* power analyses. Based on the number of invited collaborators, we anticipated a sample size of approximately 220 dyads (our higher order unit of analysis). Sensitivity analyses indicated that the minimum effect size that we could observe with an 80% power, for the proposed sample size (*n* = 220) is *d* = 0.38 [81], but we planned to report the adjusted observable effect size at 80% power following data collection. We planned to explore the main effect of condition (confirmatory) and whether this effect differed as a function of role (listener versus speaker; not hypothesized). Our estimated sample size (*n* = 220) had a power of 80% to detect the often-used benchmark of *d* = 0.50 in a one-tailed test. A one-tailed test has been argued to be convincing when combined with preregistration and *a priori* directional hypotheses [82,83], as in the case of the present research.

Our attained sample (*n* = 606) was 303 dyads in total, 606 participants. Sensitivity analysis indicated that the smallest effect size that this sample can detect with an 80% power, one-tailed test is *d* = 0.20 [83]. The intraclass correlation coefficients of the positivity resonance, authenticity and state anxiety dependent variables were 0.00, 0.41 and 0.02, respectively, which is below the 0.45 threshold for a consequential non-independence [84].

The high-quality listening condition consisted of 70% female, 29% male and 1% another gender (including non-binary). Participants identified as British (English/Welsh/Scottish/Northern Irish; 52%) and any other white background (6% including 1% Irish). Other ethnic groups represented included Arab (5%), Asian or Asian British (15%), Black, African, Caribbean or Black British (9%) and mixed or multiple ethnical groups (6%).

The moderate-quality listening condition consisted of 71% female, 28% male and 1% another gender (including trans-woman). Participants identified as British (English/Welsh/Scottish/Northern Irish; 54%) and any other white background (5% including 1% Irish). Other ethnic groups represented included Arab (4%), Asian or Asian British (20%), Black, African, Caribbean or Black British (6%) and mixed or multiple ethnic groups (7%).

In terms of disabilities, 2% of participants in both conditions reported issues with vision, and 1% with hearing (upon examining the data further all were in the role of speaker). As for learning disabilities, 2% of the high-quality listening condition, and 5% of the moderate-quality listening condition presented with difficulties including dyslexia, attention deficit hyperactivity disorder and recovering from a concussion. In each condition, 5% of participants reported social or behavioural disabilities. Four participants (0.6% per condition) could not speak English very well (also in the role of speaker).

### 4.3. Exclusion criteria

Dyads were excluded from analyses if they (i) Talked about the manipulation itself, and/or (ii) Engaged in unrelated conversation in more than 30% of the chat (2 min). The latter was done to ensure we had at least 4 min of listening relevant conversation to evaluate to ensure that the majority of the conversation had been on the topic of focus. These two qualities were determined by researchers coding the conversation videos. We also added an attention check item to the surveys completed after the conversation, ‘please mark 5 for this question’, and excluded any responses that failed this attention check.

## 5. Research design and procedure

**Figure 2.**
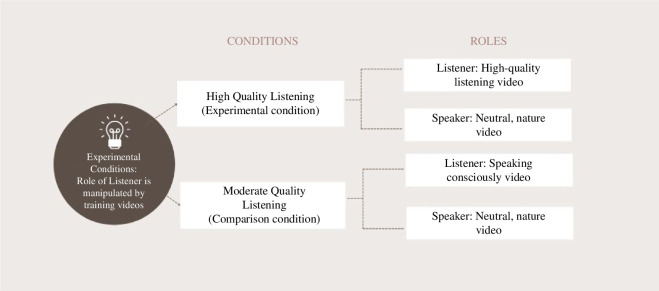
Experimental versus comparison conditions.

This study used a randomized 2 × 2 between-participant experimental design crossing listening quality (high versus moderate) and role (speaker versus listener). Those in the role of ‘listener’ were trained in listening by watching a short training video; receiving high-quality listening training or moderate-quality listening training as a comparison. During this time, all those in the role of ‘speaker’ watched a neutral nature video of the same length (refer to figure 2).

### 5.1. Procedure

We followed the procedure as described in the registered report. Following a briefing and consent process including consent to video-record the conversation, participants were paired with a conversation partner with whom they were not familiar. Each individual within the dyad was randomly assigned to one of two listening training conditions (high-quality listening training or moderate-quality listening training - comparison condition), and one of two roles (listener; speaker).

Student researchers introduced the pairs to one another and coordinated the listening interaction. Participants met via video-conferencing using Microsoft Teams using the university’s secure IT infrastructure (allowing flexibility for restrictions due to social distancing needs). Sessions were video recorded using Microsoft Stream so that interactions could be coded by student researchers (see more on this below). To maximize interaction quality via video-conferencing, guidance was provided on camera positioning (e.g. having shoulders and arms visible, the camera aligned with the screen so that eye contact is level and maintained) and lighting, as well as setting up the interaction in a private space where there are unlikely to be interruptions or distractions to ensure optimal conditions for virtual communication (see: https://youtu.be/5eQWag1lkR8). Participants were discouraged from using smartphones for video-conferencing due to limited screen size and quality of video interaction. Where participants did not have access to video-conferencing facilities or where supervisors preferred it, a lab was available on the university campus where in-person interactions could take place and be video-recorded.

The listening manipulation, shown only to participants in the role of ‘listener’, was delivered through a validated set of stimuli: (i) a training video describing high-quality listening, or (ii) a comparison condition video intended to invoke moderate-quality listening by encouraging the participant to focus on ‘conscious speaking’ (see electronic supplementary material, appendix 1 for full details of videos). Participants in the role of ‘speaker’ watched a neutral unrelated film for the same period of time. These videos are described in more detail below under §6.1.

Participants were then guided by student researchers to engage in a short conversation about character strengths for 6 min. This time was selected based on previous experiments with trained researchers lasting 8–10 min that balanced time to discuss and listen well with the ability of participants to maintain a natural flow of conversation in this lab paradigm [80,85]. In this study, we shortened the conversation because the current study’s listeners were not trained to extend the conversation deliberately as in previous experiments. Thus, this timing allowed sufficient time for participants to engage in an active and aware manner, but not so much time that the flow of conversation was exhausted. Conversation instructions, as well as the training videos, made it clear that silence was acceptable and encouraged in order to reduce the likelihood of feelings of awkwardness during the listening interactions.

### 5.2. Discussing strengths

We adapted scripts from the study on strengths awareness [70], as the script has been found to successfully manipulate a strengths condition in comparison to a weakness and neutral conditions. We adapted the description of strengths to align with the definition of VIA character strengths [61] and for a speaking, rather than writing, exercise:

We are interested in gaining insight into your personal character strengths. In other words, we would like to understand what virtuous characteristics you feel most energized by and that feel most authentic to who you are. Refer to the VIA character strengths information sheet for a list of character strengths (see study registration on OSF for materials). Think back over the past week and talk about your personal character strengths, relating them to experiences that you had during the past week. Be as specific and detailed as you can when you describe the experiences in which your strengths manifested. There is no correct answer to this question, and you are not being assessed on your speech in any way. Feel free to speak whatever comes to mind, and know that it is normal and acceptable to have periods of silence to allow time for thinking while you express yourself [70].

Participants completed the follow-up scales described below immediately following the interaction. Student researchers had the option to further assess listening quality by rating behavioural observations or analysing transcripts from the video recordings.

## 6. Materials

### 6.1. Listening training

Training for participants in the role of listener consisted of a set of two, short expert training videos of approx. 13 min in length (see electronic supplementary material, appendix 1 for scripts). The moderate-quality listening training comparison condition mirrored the same format and content structure as the high-quality listening training condition to reduce possible confounding effects, training listener participants to *speak consciously* rather than focus on high-quality listening. Participants in the role of speaker viewed a nature documentary of a comparable length, selected to be neutral to interpersonal contexts.

Training videos were created and validated by the researchers as part of ongoing research into listening, specifically listening training [86]. Research into the central themes of practitioner listening training informed the content of the high-quality listening condition training videos, which had an academic and professional tone including suggestions on how to listen well from listening experts (see electronic supplementary material, appendix 1 for further details of listening video development and validation).

### 6.2. Measures

#### 6.2.1. Overall approach to study scales

We applied a scale ranging from 1 (*not at all*) to 7 (*extremely*) for consistency across the questionnaires unless an alternative is indicated in the scale description below, and because 7-point Likert-type scales have been shown to provide higher internal consistency and test-retest reliability than scales with fewer points [87]. Where possible, we opted for brief versions of the scale to respect participants’ time. We tested for internal reliability for all questionnaires with three or more items to establish internal reliability of *α* ≥ 0.70. We then averaged all items on a scale after reversing items as appropriate. All scales met the threshold of *α* ≥ 0.70 (see table 2). Therefore, we did not need to implement the pre-planned strategy outlined in part one of the registered report of excluding the lowest-loading items one by one until the threshold was achieved. Where relevant, items were phrased accordingly to align with either the speaker or listener in the dyad.

#### 6.2.2. Listening quality (manipulation check)

We sought to establish a high-quality listening experimental condition. The Facilitating Listening Scale (FLS [88]) measures perceptions, and attributions of consequences of listening behaviours by a conversation partner as perceived by a speaker. Items have been developed from existing measures and theories on listening resulting in nine factors and scales. We leveraged the Constructive-Listening Behaviour subscale only consisting of 10 items (previous α consistently exceeded 0.90 [88]) to measure the perception of high-quality listening by the speaker on a continuum from poor to high quality. We applied an 11-point Likert-type scale as suggested by the authors to reduce the effects of scale coarseness (*cf*. [89]). Items include ‘pays close attention to what I say’ and ‘gives me time and space to talk’. We adapted the scale so that listeners could also self-report on their own quality of listening (speaker *α* = 0.87; listener *α* = 0.81).

#### 6.2.3. Positivity resonance

We used the seven-item Episode-Level Positivity Resonance Scale [90] to measure facets of positivity resonance (shared positivity, mutual care and concern, behavioural and biological synchrony) that occurred during the interaction (previous *α* = 0.96–0.97). Example questions are, ‘Did you experience a mutual sense of warmth and concern toward the listener?’, ‘Did thoughts and feelings flow with ease between you and the conversation partner?’ and, ‘Did you feel in sync with the conversation partner’? The following stem was applied: ‘Considering only the time during this episode when you were interacting with your conversation partner, for what proportion of the time. . .’ Items were answered on a scale from 0 to 100 (speaker *α* = 0.93; listener *α* = 0.93).

#### 6.2.4. (Perceived) authentic expression

The Authentic and Inauthentic Expression Scale (AIES [43]) measures two aspects of self-expression; authentic and inauthentic with four items each (previous *α* authentic subscale = 0.96 and *α* inauthentic subscale = 0.92 [43]). In the current study, we only used the authentic expression scale, an approach the authors [43] recommend to take when more suitable for the study. Items included ‘I express my real thoughts and feelings to others’ (speaker *α* = 0.87; listener *α* = 0.91).

#### 6.2.5. State anxiety

We used an adapted version of the Short Version of the Spielberger State-Trait Anxiety Inventory (STAIS−5 [91]), which has shown high reliability, *α* = 0.91, and high correlations with the original scales and other well-established comparable measures. Authors excluded reverse-scored items from the original Spielberger scale [92] to improve reliability, internal consistency and validity, then applied Item Response Theory analyses to reduce the number of items per scale according to the optimal threshold (*α* > 0.17) for discrimination ability. While a total of nine items met the threshold, the STAIS−5 consists of the five highest-scoring items. Not all items are relevant for our context (e.g. frightened, upset, confused); we therefore retained two (nervous, jittery) and selected three alternative items from the original nine that met the threshold that would be sensitive to the aspects of anxiety experienced during a brief conversation about a neutral to positive topic (tense, strained, and worried), (speaker *α* = 0.90; listener *α* = 0.90).

#### 6.2.6. Positive and negative experiences

The scale of positive and negative experience (SPANE, hereafter referred to as positive affect [93]) has 12 items, with six items focusing on positive affect and six focusing on negative affect, rated on a Likert-type scale ranging from *very rarely or never* to *very often or always*. Positive and negative affect scales are scored separately, then negative affect is subtracted from positive affect for a relative score. Psychometric statistics for the scale have previously shown acceptable internal consistency (*α* = 0.87 and temporal stability = 0.62). The SPANE correlates strongly with PANAS as well as other similar, short measures of affect [93] (positive affect speaker *α* = 0.87, listener *α* = 0.87; negative affect speaker *α* = 0.80, listener *α* = 0.84).

#### 6.2.7. Behavioural intention measures

We adapted items from [94] to measure the likelihood of participants to either use their strengths (for speakers; *α* = 0.89) or continue engaging in listening (for listeners; *α* = 0.81). Behavioural intention (e.g. *‘I intend to…*’) and self-prediction (*‘How likely is it that you will…*’) items have been shown to relate to subsequent behaviour [95]. We adopted both self-prediction and behavioural intention items, as self-prediction items take into account constraints to performing behaviours. We employed a subjective probability scale from 1 (*not at all likely*) to 7 (*extremely likely*) as this has been shown to be more suitable than forced-choice measures for behavioural intention [96]. Items were as follows:

1. Intention to use strengths (speakers):
  - ‘Now that you have discussed your character strengths, how likely is it that you will use and apply your character strengths?’
  - ‘I intend to use and apply my character strengths that I spoke about today, as I engage in life and work activities in the future’.
2. Intention to continue practising listening (listeners):
  - ’Now that you have practised being a listener, how likely is it that you will continue practising the listening skills you applied in today’s interaction?’
  - ‘I intend to continue listening in the way I have today when I am engaging with people in future conversations.’

## 7. Listening observer ratings

To triangulate measures of listening quality by participants, we included an observational measure of listening quality which was intended to be undertaken by student researchers as they played back a recording of the video conversation. Due to time restrictions associated with the end of the academic term, we were unable to resolve reliability issues in the initial video coding. Following editor recommendations, we re-coded the 120 videos that we retained from the students with two new and trained coders to allow us to calculate an independent observer’s score as originally planned. Mirroring the method of listening observations ratings in [97], we reviewed recordings of the last 4 min of the interaction—where high-quality listening was expected to take place—and rated high-quality listening behaviours. The new coding team rated the videos in blocks of 30-s clips. As indicated in stage one of the registered report, a portion of videos were double-coded so that inter-rater reliability (IRR) could be calculated between coders (aiming for a minimum of 0.41—moderate agreement [98]). When reliability fell below 0.61—substantial agreement, coders discussed discrepancies and revisited ratings to ensure that the rating scale had been interpreted consistently. As we were exploring the full range of high-quality listening behaviours, coders rated more than just active listening statements as was done by [97] and covered the three core components of good listening (body language, verbal behaviours and positive intentions) as outlined earlier in our introduction. Behavioural indicators are outlined below and were included in an observation score sheet.

1) Body language:

— Maintains appropriate eye contact with the speaker (without staring)
— Posture is open and either leaning in or facing towards the speaker
— Facial expression is neutral or positive, and conveys openness
— Head nodding

2) Verbal behaviours:

— Summarizes the speaker’s content (e.g. paraphrasing or in speaker’s words)
— Asks open-ended questions to clarify understanding or show interest
— Uses verbal cues (e.g. *‘uh huh’* or *‘mmm’* etc.)
— Positive or neutral tone of voice

3) Positive intentions:

— Offers validation (e.g. ‘*Thank you for sharing*’ or ‘*That sounds interestin*g’)
— Uses soft hedging phrases such as *‘perhaps’* or *‘might’*
— Allows silence, time and space for speaker to express themselves fully (e.g. does not speak over them or fill in silences when the speaker is obviously thinking)

Since body language could not be rated by frequency, coders allocated one point per indicator per 30 second block but deducted the point if any of these were violated per 30-s block (e.g. listener looks away for a noticeable period of time—0 points for the 30-s block, or adopts a judgemental tone of voice in one of their responses—0 points for the 30-s block, nods head 3 or 4 times in the 30-s block—1 point for the 30-s block, facial expression remains neutral or positive across the 30-s block, 1 point). The remaining verbal behaviours and positive intention behaviours were rated by frequency of occurrence per 30-s block. Scores for each section were summed such that the higher the overall score, the better the listening quality.

## 8. Analysis strategy

The following recaps the planned analysis steps as stipulated in stage one of the registered report.

### 8.1. Preliminary tests

#### 8.1.1. Collinearity

Feedback from reviewers in the first stage of the registered report raised a point that the three dependent variables of listening (state anxiety, positivity resonance and authenticity) could be correlated at least modestly (e.g. [99]). Therefore, we planned to empirically distinguish between the three variables before performing our analyses by measuring the scale score correlations and comparing point estimates against a cut-off of *r* = 0.70 (a level we have judged as sufficient for this study based on a plausible conceptual distinction between the variables [100]). Where correlations exceed *r* = 0.70 between two or more of the variables, we would accept that they are not empirically distinguishable, and plan to average the scores and treat them as reflecting one multi-faceted construct.

#### 8.1.2. Manipulation checks

Manipulation checks were planned to determine listening quality as the independent variable across conditions. These included triangulated measures of listening quality; including a self-report by the listener, speaker-report and independent observer evaluations. For *speakers*, a manipulation check would involve a between-subjects analysis of variance (ANOVA) predicting perceived listening from the high-quality listening versus moderate-quality listening comparison condition contrast. For *listeners,* the high-quality listening versus comparison condition would predict the perception of their own listening. A final test would predict listening behaviours as recorded by independent observers from these two conditions. No covariates were defined for these models. We anticipated the manipulation would be entirely successful in effecting listening providing that all three models showed significant condition effects at *p* < 0.05. Along with statistical significance, we planned to report effect sizes and their confidence intervals. The manipulation would be interpreted to be partially successful if the condition effects any, but not all, of the outcomes at *p* < 0.05.

*Hypothesis 1* was planned to be tested with a multivariate analysis of variance (MANOVA) predicting simultaneously the three immediate, conversation-specific outcomes of authenticity, positivity resonance and anxiety from a 2 (between-subjects: condition: high-quality listening versus comparison condition) × 2 (between-subjects: role: listener versus speaker). To evaluate all possibilities, we planned to test but did not anticipate, a two-way interaction effect because we expected that speakers and listeners would benefit similarly in terms of their authenticity, positivity resonance and anxiety when the listener engages in high-quality listening. In short, hypothesis 1 was tested through the main effect of condition across roles. If we observed a statistically significant condition main effect at *p* < 0.05, we planned to interpret hypothesis 1 as being partially supported; if we observed condition main effects on all three outcomes, we planned to interpret hypothesis 1 as being fully supported. Despite the focus on main effects, if we did find an unexpected two-way interaction at a statistical significance of *p* < 0.05, we planned to test simple slopes for listener and speaker, separately, for the outcome that showed the interaction effect.

*Hypothesis 2a* was planned to be tested with a between-subjects ANOVA similar to that used to test hypothesis 1 but including only speakers. For speakers only, the condition would predict the intention to use strengths as an outcome. If we observed a statistically significant effect of condition at *p* < 0.05, we would interpret hypothesis 2a as being supported.

*Hypothesis 2b* would be tested with a between-subjects ANOVA to align with hypothesis 1 but include only listeners. For listeners only, the condition would predict the intention to continue listening well as an outcome. If we observed a statistically significant effect of condition at *p* < 0.05, we would interpret hypothesis 2b as being supported.

*Hypothesis 3a* would be tested with model 4 in PROCESS [101] using 5000 bootstrapped samples. For *speakers* only, defining condition as a predictor, the three mediators (authenticity, positivity resonance, anxiety) simultaneously and intention to use strengths as an outcome. We would therefore examine mediators in competition for variance in the outcome. We would interpret indirect effects through each of the three mediators, understanding them to be present if they are statistically significant at *p* < 0.05.

*Hypothesis 3b* would be tested using the same PROCESS approach for *listeners* only, defining condition as a predictor, the three mediators (authenticity, positivity resonance, anxiety) simultaneously, and intention to continue listening as an outcome. We would therefore examine mediators in competition for a variance on the listener’s intention to continue listening and understand indirect effects to be evident providing they are significant at *p* < 0.05.

## 9. Results

Results of the study are described below and followed our phase one registered report plan except that the coding of listening by independent observers was not performed by the student researchers as proposed, but by a separate team of coders due to student–researcher timeframe restrictions. All data are available on our Open Science Framework (OSF) page: https://osf.io/q2bgr/?view_only. The software SPSS Statistics Version 28 was used to perform analyses.

In table 1, we present the means and s.d. for each variable. Means for listening quality were above the mid-point for both the high-quality and moderate-quality listening conditions, indicating that there was generally a high quality of listening in both conditions, yet there appeared to be a greater perceived difference in listening quality by the speaker across conditions. Positivity resonance, authenticity and behavioural intention measures were also above the mid-point, and state anxiety was below across both conditions.

**Table 1.** Descriptive statistics for conditions and variables. Note. Difference between means: *p < 0.05, **p < 0.001. ∞, frequency of observed behaviours. CS, character strengths.

variables	scale range	high-quality		moderate-quality	
		*M*	*SD*	*M*	*SD*
listening (self-report)	1–11	8.92*	1.06	8.53*	1.23
speaker (perceived listening)	1–11	9.42**	1.09	8.96**	1.38
observed listening	∞	55.63	8.43	52.72	11.49
state anxiety	1–7	2.06	1.16	2.19	1.28
positivity resonance	0–100	74.95*	16.22	71.80*	15.96
authenticity	1–7	5.83	1.18	5.77	1.19
positive affect	1–7	4.08	1.41	4.04	1.47
intention to continue using CS	1–7	5.61	0.97	5.63	1.11
intention to continue listening	1–7	5.85*	0.95	5.62*	0.98

### 9.1. Collinearity

Table 2 presents the correlations between study variables. As we did not find correlations exceeding *r* = 0.70 between two or more of the variables, we could accept that the dependent variables are empirically distinguishable and sufficiently distinct to be modelled simultaneously. Positivity resonance was strongly correlated with positive affect (*r =* 0.61). State anxiety also had a strong negative correlation with positive affect (*r =* −0.58) and a weaker negative correlation with positivity resonance (*r =* −0.31) and authenticity (*r =* −0.20). We did not include positive affect as a main outcome in our model, however, theoretically it is a component of positivity resonance.

**Table 2.** Correlations and Cronbach’s alpha of study variables. Note. **p < 0.001 level. *α* in brackets.

variables	1	2	3	4	5	6	7
listener (self-report)	(0.81)						
speaker (perceived listening)	0.33**	(0.87)					
state anxiety	−0.30**	−0.16**	(0.90)				
positivity resonance	0.39**	0.53**	−0.31**	(0.93)			
authenticity	0.31**	0.30**	−0.20**	0.42**	(0.91)		
positive affect	0.37**	0.46**	−0.58**	0.61**	0.39**	(0.87)	
intention to continue strengths	0.05	0.27**	−0.28**	0.32**	0.47**	0.37**	(0.89)
intention to continue listening	0.42**	0.13**	−0.17**	0.38**	0.27**	0.35**	0.06

Other important results include that self-reported listening and perceived listening as rated by speakers is weakly correlated (*r =* 0.33). Furthermore, self-reported listening was moderately correlated with positivity resonance (*r =* 0.39) and with an intention to continue listening after the conversation (*r =* 0.42); and weakly correlated with positive affect (*r =* 0.37), authenticity (*r =* 0.31) and lower state anxiety (*r =* −0.30).

Speakers’ perceived listening was strongly positively correlated with positivity resonance (*r =* 0.53) and positive affect (*r =* 0.46) and showed weaker positive correlations with authenticity (*r* = 0.30) and intention to continue using character strengths (*r* = 0.27) following the conversations.

The speaker’s evaluation of listening showed a very weak relationship with an intention to continue listening by the listener (*r* = 0.13). The listener’s self-reported quality of listening showed little to no relationship with the speaker’s intention to continue using character strengths (*r* = 0.05); and finally, intention to continue listening and to continue using character strengths also showed little to no relationship (*r* = 0.06).

## 10. Listening quality manipulation check

A between-subjects ANOVA for speakers’ perceived listening showed a significant effect of condition on listening quality, *F*(1, 293) = 10.34, *p* < 0.001, Cohen’s *d* = 0.38, 95% CI [0.14, 0.61], showing a positive difference between the high-quality listening versus the moderate-quality listening comparison condition. A condition effect was also present for listeners’ self-rating of their own listening between the high-quality and the moderate-quality listening conditions, *F*(1, 303) = 8.92, *p* = 0.003, Cohen’s *d* = 0.34, 95% CI [0.17, 0.57]. Results support that participants who were trained with the high-quality listening training video were perceived by both the speaker and the listener (self) to demonstrate higher quality listening during the experimental conversation with a small effect size.

### 10.1. Listening observation ratings

A total of 130 videos were coded for listening quality by two coders (high-quality: *n* = 66; moderate-quality: *n* = 64). The intraclass correlation coefficient calculated using a two-way mixed, average measures ICC [102] revealed a reasonable level of agreement between coders, ICC = 0.802, *p* < 0.001, *α* = 0.86. Observer ratings between the conditions showed no significant difference in the total listening quality, *p* = 0.103.

Table 3 reports descriptive statistics and results of the between-subjects comparison of means between conditions for the total listening score, and the three sub-component scores of listening observed. A significant difference for the subscale of body language only, *p* = 0.037 was evident.

**Table 3.** Listening observation scores (total and subscales). Note. HQ, high-quality listening condition; MQ, moderate-quality listening (comparison) condition. Difference between means: *p < 0.05.

listening observations	*condition*	*N*	*M*	*s.d.*	*t*	*p*	*d*	Cl (95%)
total listening	HQ	66	55.63	8.43	1.64	0.103	0.29	[−0.06, 0.64]
	MQ	64	52.72	11.49				
body language	HQ	66	36.20	3.39	2.11	0.037*	0.37	[−0.02, 0.72]
	MQ	64	34.66	4.82				
verbal behaviour	HQ	66	12.13	5.65	0.97	0.334	0.17	[−0.17, 0.52]
	MQ	64	11.08	6.63				
positive intention	HQ	66	7.43	3.01	0.95	0.342	0.17	[−0.18, 0.51 ]
	MQ	64	6.91	3.25				

Overall, the listening manipulation was successful across two (self-report and speaker perception) out of three of our triangulated measures of the independent variable, thus we deem the listening manipulation as having been partially successful.

*Hypothesis 1* was tested with a two-way MANOVA predicting three immediate, conversation-specific outcomes of authenticity, positivity resonance, and anxiety from a 2 (between-subjects: condition: high-quality listening versus comparison condition) × 2 (between-subjects: role: listener versus speaker). Statistics are presented in table 4: Tests of between-subjects effects by condition showed a significant, positive difference for positivity resonance experienced by both the listener and speaker in the high-quality listening condition compared with the moderate-quality listening condition with a small effect size. No effects were present between conditions for state anxiety or authenticity, therefore, *H*_1_ was partially supported.

**Table 4.** *F* values, *p* values, Cohen’s *d*, confidence intervals for each of the dependent variables hypothesized. Note. *p < 0.05.

dependent variables	*F*	*df*	*p*_(one-tailed)_	*d*	CI (95%)
state anxiety	1.82	596	0.089	−0.11	[−0.27, 0.05]
positivity resonance	5.70	596	0.009*	0.20	[0.04, 0.36]
authenticity	0.45	596	0.252	0.05	[−0.11, 0.21]
intention to continue CS	0.03	293	0.433	−0.02	[−0.25, 0.21]
intention to continue listening	4.64	302	0.016*	0.25	[0.02, 0.47]

While the test for an interaction effect between role and condition was not significant for any of the dependent variables: state anxiety *F*(1, 596) = 0.88, *p* = 0.348; positivity resonance *F*(1, 596) = 0.01, *p* = 0.927; authenticity *F*(1, 596) = 1.00, *p* = 0.318; it is worth noting that a main effect of role (listener or speaker) predicting authenticity was evident, *F*(1, 596) = 39.84, *p* < 0.001, *d* = 0.52 (medium effect size); thus speakers appeared to experience greater authenticity than listeners.

*Hypotheses 2a and 2b* results are presented in table 4, and were tested with a between-subjects ANOVA. Results supported that condition predicted the listener’s intention to continue listening (*H*_2b_) with a small effect size. Condition did not predict the speakers’ intention to continue using character strengths (*H*_2a_). Results suggest that listeners in the high-quality listening condition were motivated to continue their listening behaviour following the interaction. The high-quality listening condition did not predict the speakers’ intention to continue applying their character strengths, however.

*Hypotheses 3a and 3b (confirmatory analyses)* were tested with model 4 in PROCESS [101] using 5000 bootstrapped samples. The model for *H*_3a_ (including total effect, direct effect and indirect effects) was not supported, therefore condition did not predict *speakers’* intention to continue using character strengths via the three mediators (authenticity, positivity resonance, anxiety) simultaneously.

For *H*_3b_, the total effect of the model; that condition would predict listeners’ intention to continue listening was significant, *b* = −0.24, s.e. = 0.11, *t* = −2.15, *p* = 0.032, 95% CI [–0.46,–0.02] consistent with our results for *H*_2b_. In contrast, the direct effect of condition to intention to continue listening: *b* = −0.18, s.e. = 0.10, *t* = −1.73, *p* = 0.085, 95% CI [−0.38, 0.02] and indirect effects of positivity resonance, *b* = −0.06, s.e. = 0.03, 95% CI [−0.13, 0.00]; authenticity *b* = 0.00, s.e. = 0.02, 95% CI [−0.04, 0.04]; and state anxiety *b* = 0.00, s.e. = 0.01, 95% CI [−0.03, 0.02] were not statistically significant. This indicates that the effect (behavioural intention) was not a direct result of the condition, nor mediated from condition by authenticity, positivity resonance and anxiety. Thus, *H*_3b_ was also not supported.

## 11. Exploratory analyses

To explore the contradictory finding of *H*_3b_ further, we performed auxiliary mediation analyses (tested with model 4 in PROCESS using 5000 bootstrapped samples [101]). Since our listening manipulation was only partially successful, we replaced condition as the predictor in the models, with speaker’s perception of listening for *H*_3a_, and self-reported listening for *H*_3b,_ since conceptually, the individual’s own perception of listening quality (*x*) would be most important for their own motivation to continue the behaviour (*y*).

### 11.1. Intention to continue using strengths

**Figure 3.**
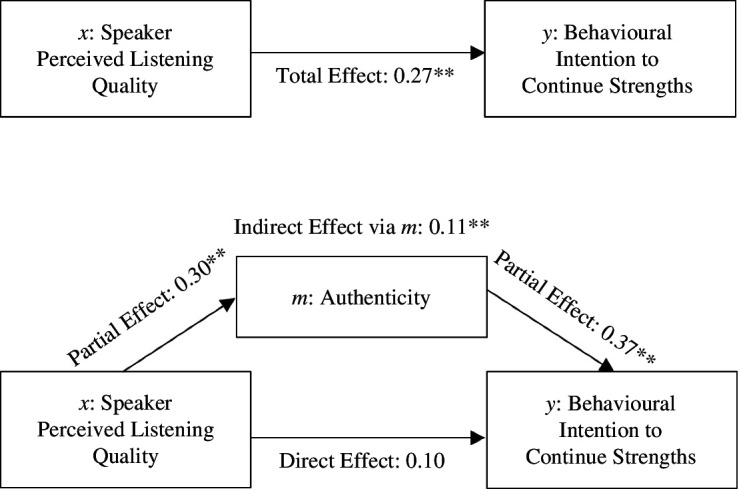
Exploratory mediation analyses for the pathway from speaker-perceived listening (*x*) to behavioural intention to continue strengths (*y*) via authenticity (*m*). Note. Standardized pathways. **p < 0.001.

For *H*_3a_, the total effect of the revised model (figure 3) was significant: *b* = 0.22, s.e. = 0.05, *t* = 4.86, *p* < 0.001 [0.13, 0.31]. The direct effect of *x* on *y* was not significant: *b* = 0.08, s.e. = 0.05, *t* = 1.66, *p* = 0.097 [−0.02, 0.18]. Speaker perceptions of listening quality showed significant partial effects on all three dependent variables (state anxiety, *b* = 0.16, s.e. = 0.06, *t* = −2.79, *p* = 0.006 [−0.26, −0.05], positivity resonance *b* = 6.71, s.e. = 0.63, *t* = 10.68, *p* < 0.001 [5.47, 7.95], and authenticity *b* = 0.20, s.e. = 0.04, *t* = 5.34, *p* < 0.001 [0.13, 0.28]). Only the indirect effect with authenticity as a mediator to behavioural intention to continue using strengths was statistically significant, *b* = 0.09, s.e. = 0.03, [0.04, 0.16]. The indirect effects for state anxiety *b* = 0.02, *SE* = 0.01, [−0.00, 0.04] and positivity resonance *b* = 0.04, s.e. = 0.03 [−0.02, 0.09] were not statistically significant, and thus only authenticity appeared to mediate the effects. Thus, authenticity accounted for approximately 41% of the variance of the effect of speaker-perceived listening quality on intention to continue using strengths, however, perceived listening quality did not have a significant direct effect on behavioural intention.

### 11.2. Intention to continue listening

**Figure 4.**
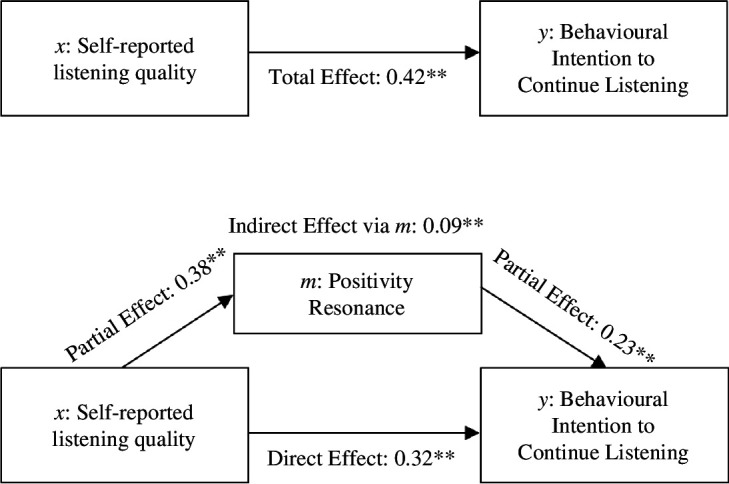
Exploratory mediation analyses for the pathway from self-reported listening (*x*) to behavioural intention to continue listening (*y*) via positivity resonance (*m*). Note. Standardized pathways. **p < 0.001.

For *H*_3b_, the total effect of the revised model (figure 4) was significant: *b* = 0.35, s.e. = 0.04, *t* = 8.05, *p* < 0.001 [0.27, 0.44], as was the direct effect of *x* on *y*: *b* = 0.26, s.e. = 0.05, *t* = 5.52, *p* < 0.001 [0.17, 0.35]. Self-reported listening quality reported a significant partial effect on all three dependent variables at ps < 0.001 (state anxiety, *b* = −0.30, s.e. = 0.06, *t* = −5.23, *p* < 0.001 [−0.41, −0.18], positivity resonance *b* = 5.37, s.e. = 0.75, *t* = 7.16, *p* < 0.001 [3.89, 6.84], and authenticity *b* = 0.36, s.e. = 0.06, *t* = 5.62, *p* < 0.001 [0.23, 0.49]). However, only the indirect effect with positivity resonance as a mediator was statistically significant *b* = 0.07, s.e. = 0.03 [0.02, 0.16]. The indirect effects for state anxiety *b* = 0.00, s.e. = 0.02 [−0.03, 0.03] and authenticity *b* = 0.02, s.e. = 0.02 [−0.02, 0.06] were not statistically significant, and thus only positivity resonance appeared to mediate the effects. Positivity resonance accounted for approximately 21% of the variance of the effect of self-reported listening quality on intention to continue listening, and 76% of the variance appears to be accounted for directly by self-reported listening.

## 12. General discussion

Speakers can experience a range of intrapersonal and interpersonal benefits from being listened to well, but we know less about the effects on the listener. The current experiment sought to explore the effects of high-quality listening on both the speaker and listener, in a unique experiment that involved a naturalistic conversation between interlocutors thus creating greater ecological validity. We explored the consequences of a positive conversation about character strengths, allowing us to estimate the benefits of high-quality listening over and above the known benefits of discussing character strengths [70–72]. The outcomes of the hypotheses tested in our study showed mixed findings overall for the effects of high-quality listening during the conversation. We found that of the three potential immediate outcomes of listening that we measured: namely, positivity resonance, authenticity and state anxiety—positivity resonance was the only significant outcome benefited after listeners within the dyad received a brief listening training. The effect size of listening benefits to positivity resonance was small but benefits were attained across both speakers and their listeners. High-quality listening therefore seemed to have supported feelings of shared positive affect, mutual care and concern, and behavioural and biological synchrony [46,47] between both the speaker and the listener. Furthermore, we identified a second benefit of the listening condition such that listeners in the high-quality listening condition were further motivated to listen in this way to conversation partners in the future; notably, this desire to continue behaving in ways consistent with the conversation’s aims did not extend to speakers using their character strengths.

Here, we built on a literature that finds that conversing about character strengths can elicit positive outcomes for the speaker [4,6]; our findings suggested that positivity resonance can be attributed to high-quality listening within such otherwise, still quite beneficial conversations. Positivity resonance has been conceptualized as a dyadic experience, shared between two members of a conversation. Our findings supported this view, suggesting that positive benefits from high-quality listening can benefit *both* the listener as well as the speaker. Though these findings are preliminary and based on statistically significant but relatively small differences in listening across the two conditions tested, it is worth noting the benefits of listening training were not similarly observed for our intrapersonal outcomes of authenticity and reduced anxiety, suggesting that the relational nature of listening may be the most prominent effect of high-quality listening and supporting conceptualizations that listening may bring about a sense of togetherness that is its most proximal and powerful outcome [32]. Positivity resonance can be experienced between strangers in a fleeting moment, where both individuals co-experience positive emotion, express care towards one another, and share behavioural and biological synchronicity—but it is the repeated experience of positivity resonance that builds toward a more complete, relational concept of love or intimacy [46].

Links between high-quality listening and positivity resonance can inform conversations that aim to generate specific outcomes for both the speaker and listener. These conversations include professional helping conversations that rely on listening as a core skill, for example, counselling, therapy and coaching [12,35–37]. First, the findings are consistent with early theories on counselling and listening by Carl Rogers [1], who posits the positive relational effects on individuals when they experience the non-judgemental and caring space created by good listeners [52]. Similar to broaden and build theory [47], it is believed that positivity resonance can enhance a person’s capacity to think more openly and broadly [103]; our findings here suggested that high-quality listening—through downstream effects of positivity resonance—has the potential to improve both a speaker and listener’s capacity to think creatively, solve problems [47,104], and support wellbeing, with further potential of mitigating depression, illness and loneliness [90]. However, these conclusions should be drawn with caution, as positivity resonance did not seem to further benefit speakers in our current study.

Also applicable to these professions is that our findings were generalized across speakers and their listeners. Professionals themselves are at risk for mental health costs, especially when discussing potentially confronting or traumatic topics [10]. Understanding the listeners’ experience of positivity resonance as well as the speakers’ suggests that high-quality listening has the potential to minimize or ‘buffer’ practitioners from the risk of professional burnout [105]. Since our results suggest that receiving listening training predicted the listener’s desire to continue listening, the listener likely perceived a benefit from their high-quality listening. This provides indirect evidence for listening well as a protective factor for those within listening professions, as well as for those whom they serve.

Confirmatory analyses of our mediation models exploring the effects of condition on the dependent variables as a mediator for intention to continue listening or continue using strengths were contradictory to results obtained in the study, producing null results. As our listening manipulation was only partially successful, exploratory analyses which replaced the listening condition as the predictor within the mediation model, and instead modelled listener*-*reported listening as a predictor of listeners’ own intention to listen, and speaker-reported listening as a predictor of speakers’ intention to utilize their character strengths produced further insights. Analyses support that at the least, perceptions of listening may play an important role in conversations deemed satisfying and engaging, specifically in inspiring conversants’ continued engagement. Positivity resonance mediated these downstream benefits for listeners with intention to continue listening, suggesting tentatively that interpersonal connection may play an important role in building satisfying or productive relationships. An unexpected finding of this exploratory model, which sought to reproduce condition effects with a more sensitive predictor (namely, self-reported listening quality), was that state authenticity (rather than positivity resonance) mediated the effects from speaker-perceived listening to intention to continue using strengths. This tentative result is explored further below.

## 13. Developing a science of listening

Using a novel approach within this field, we manipulated listening quality through a brief listening training delivered by video to participants who otherwise did not receive training as a methodological improvement to previous research attempts to manipulate listening [24,78]. Our manipulation checks suggested that laypeople could be, to some extent, trained to listen well after a brief, 13-min listening training video. Participants who watched the high-quality listening training video were perceived—at least by themselves and the speaker, to have demonstrated significantly better listening than those in the comparison condition. However, the effect sizes of the manipulation were quite small, and not supported by observer coding of the videos.

The small but significant effects of training on both listener-reported and speaker-reported listening informs literatures attempting to understand and improve listening within conversations. In the past, there have been mixed findings in the success of listening training, particularly shorter length training [106] and in achieving significant differences in perceived listening by others over self-ratings [97,107]. Our study findings support that brief listening training can show promise in supporting high-quality listening behaviour that has downstream effects on perceived listening by speakers, specifically in the short term and during a short interaction. While we are not suggesting that learning to listen well is a process that can be a ‘shortcut’ (e.g. [86]), we acknowledge there may be some benefit of brief training, particularly with professionals for whom relational listening during short interactions could have significant downstream benefits, for example, doctors with patients to avoid malpractice claims [108] or teachers with students to increase the potential for academic success [109].

Our independent observer ratings—also a relatively new method in a field dominated by self-reports of listening—did not show a condition difference in listening quality. We hope future work can fine-tune and therefore advance this process to improve our understanding of listening within conversations. In the current study, correlations between the total listening scores and subscores of body language, verbal behaviour and positive intention reveal the strongest correlations with verbal behaviour: *r* = 0.84; followed by body language: *r* = 0.69 and positive intention *r* = 0.50–64 (for both coder 1 and coder 2, respectively, in our team). We suggest fine-tuning observable markers for positive intention may be a worthwhile endeavour in future studies to improve accuracy.

## 14. Limitations

As discussed above, we observed small effect sizes in both the listening manipulation and the outcome of positivity resonance. The approach to manipulating listening needs to be improved to create meaningful change in how people listen. However, the relatively weak manipulation could be explained in part by the topic being discussed, namely, character strengths, which may have been an inherently engaging topic of conversation inspiring natural listening and connection. Indeed, both conditions showed high levels of listening and positivity resonance. It may be that under conditions where high-quality listening is less likely, such as listening to views with whom one disagrees (e.g. [48,110], listening training may play a more important role. Other strategies to improve the listening manipulation might be to allow listening training participants more time to practice and embed the listening skills they have learned, for example, by leveraging goal-setting theory [111,112] and setting a ‘listening learning challenge’ prior to the conversation where they practice listening well in their day-to-day conversations (with before and after assessments to measure their listening progress).

The topic of conversation, namely, character strengths, may also explain the absence of condition effects for state anxiety and authenticity. High-quality listening did not reduce state anxiety as was hypothesized and previously observed [31,56,57], nor did high-quality listening increase levels of authenticity for either the speaker or the listener [2,44]. The topic served as useful in allowing us to isolate the relational effects of listening against the effects of character strengths in order to address this important question raised by previous researchers [7,74,75], but character strengths are already associated with increased authenticity and wellbeing [71,113,114]. Our study findings showed that relational factors (and namely, listening) did not account for these outcomes when competing against character strengths. Indeed, exploratory analyses showed that participants in the role of speaker reported greater authenticity when compared with those in the listener role, lending some, though tentative, empirical support to the relationship between feelings of authenticity and character strengths [113]. Exploratory analyses further suggested that speakers’ perception of being listened to linked well with their feelings of greater authenticity while discussing their character strengths, and this further mediated their intention to use those character strengths. Building on this and past work on strengths interventions [74], it may be worthwhile to consider the role of listening in supporting positive change in individuals.

## 15. Future research examining listening and positivity resonance

Tools such as listening training can also be applied to areas outside of constructive conversations such as character strengths. We suggest further research to explore positivity resonance as a downstream benefit of listening (and listening training) is worthwhile in several different contexts: for example, as a tool to foster relations between people who are engaging in dialogue on challenging topics such as when discussing prejudiced or polarized attitudes [80,115]. The added dimension of a positively valenced relational experience could stimulate further benefits, for example, positive emotions have been demonstrated to have a desirable effect on perceived similarities and differences between racial characteristics [116], reducing racial bias explained in part through broaden and build theory [47,104].

Other conversations include those between people who are seeking to establish stronger relationships, such as colleagues, team members or acquaintances. A particularly interesting dynamic to explore is where there exists a power difference in the relationship between two people, such as during a performance discussion between a manager and a subordinate, or teacher and a student, where both may be required to focus on problem-solving and performance outcomes. The positive affect and relational benefits experienced by both when high-quality listening (and positivity resonance) is present may enable and empower the speaker to share ideas more openly, feel more secure, and create and suggest their own ideas and solutions [117].

Finally, conversations between friends, family members and even romantic partners could benefit from high-quality listening, and positivity resonance may explain in part the support for stronger relational bonds achieved [15,51,118,119]. It may be that listening training for parents can provide positive parenting benefits that support prevention of mental illness [120,121] and stronger intimacy that will support both parents and their children to address challenges more constructively [15].

## 16. Conclusion

We know that high-quality listening has the potential to ignite self-awareness, self-exploration and broaden attitudes in individuals who experience it. However, the effects of high-quality listening on both the speaker and listener during naturalistic conversations deserved robust experimental testing. Our study found mixed support for the conclusion that both the listener and speaker can co-experience positive outcomes in a listener-speaker engagement. Listening had few downstream effects from those we hypothesized, but notably, brief training in high-quality listening appeared to result in greater perceived listening quality by the speaker and listener, even if less so by the listeners themselves. This difference was not perceived by independent observers of listening, however. The one benefit that did yield from listening was positivity resonance—a sense of mutual warmth, caring and biological synchronicity between both the speaker and the listener. Together with other emerging evidence of the associated relational and interpersonal benefits of experiencing positivity resonance, as well as high-quality listening, we suggest that high-quality listening could be an effective tool to bolster motivation, wellbeing, and particularly, positive relationships.

## Data Availability

In line with open science principles, we have uploaded datasets to the project site on Open Science Framework (OSF): [122]. Supplementary material is available online [123].
